# Retraction Note: Dishonesty is more affected by BMI status than by short-term changes in glucose

**DOI:** 10.1038/s41598-020-80528-2

**Published:** 2021-01-12

**Authors:** Eugenia Polizzi di Sorrentino, Benedikt Herrmann, Marie Claire Villeval

**Affiliations:** 1 European Commission, Joint Research Center (JRC), 21021 Ispra, Italy; 2 Institute of Cognitive Science and Technologies, National Research Center, 00185 Rome, Italy; 3 Univ Lyon, CNRS, GATE, UMR 5824, 69130 Ecully, France; 4 IZA, Bonn, Germany

Retraction of *Scientific Reports* https://doi.org/10.1038/s41598-020-68291-w, published online 22 July 2020

The Editors are retracting this Article.

After publication concerns were raised with respect to the data suggesting a significant positive association between dishonesty and obesity. Independent post-publication peer review has confirmed that this conclusion extends beyond what the data show, given the lack of overall group effects. In addition, multiple comparisons were not appropriately controlled for, and the inclusion of gender in the analysis should have been emphasized as an exploratory addendum to the main analysis. The Editors therefore no longer have confidence in the conclusions presented.

The authors disagree with the retraction.

